# EVALUATION OF ONLINE AEROBIC TRAINING AND RESISTANCE TRAINING IN OLDER ADULTS WITH MCI: A THREE-ARM RCT

**DOI:** 10.1093/geroni/igad104.2944

**Published:** 2023-12-21

## Abstract

This study aimed to evaluate the effects of online aerobic and resistance training programs in community-dwelling older adults with mild cognitive impairment. A total of 108 participants (60~87 years) were recruited, and equally randomized to aerobic group (3-5 times/week, 30-50min/section), resistance group (2 times/week, 5 to 6 exercises/section), or control group. Cognitive functions were evaluated at baseline, the 13rd week (T1) and 26th week (T2). Effects were evaluated by linear mixed effects model (LME) after controlling for covariates. Inter-individual differences on the cognitive effects were explored. After six-month intervention, 15 (13.89%) participants dropped out, and the median compliance rates were 80.77% for aerobic group and 96.15 for resistance group. LME revealed that ADAs-cog scores in aerobic group decreased by 2.14 at T1 and 1.68 at T2 more than the control group. And the ADAs-cog scores in resistance group decreased by1.43 more than the control group at T1. For executive function, the Stroop time interference in resistance group at T2 declined 11.12 seconds more than the control group, and 13.85 seconds more than the aerobic group. For memory performance, the RAVLT scores at T1 in aerobic group tended to increase 5.15 (p = 0.065) more than the control group. There existed true inter-individual differences in the responses to exercise. Compliance rates, social activity participation, cognitive function and physical function at baseline were the significant factors. Overall, our study confirmed the positive effects of both aerobic and resistance exercise on global cognition, while their effects on specific cognitive domains were different.

